# Diaspora engagement of African migrant health workers – examples from five destination countries

**DOI:** 10.3402/gha.v8.29210

**Published:** 2015-12-09

**Authors:** Silvia Wojczewski, Annelien Poppe, Kathryn Hoffmann, Wim Peersman, Oathokwa Nkomazana, Stephen Pentz, Ruth Kutalek

**Affiliations:** 1 Department of General Practice and Family Medicine, Centre of Public Health, Medical University of Vienna, Vienna, Austria; 2 Department of Family Medicine and Primary Healthcare, Ghent University, University Hospital, Ghent, Belgium; 3 Faculty of Medicine, University of Botswana, Gaborone, Botswana; 4 Department of Family Medicine, University of the Witwatersrand, Johannesburg, South Africa

**Keywords:** international migration, health-care professionals, doctors, nurses, sub-Sahara Africa

## Abstract

**Background:**

Migrant health workers fill care gaps in their destination countries, but they also actively engage in improving living conditions for people of their countries of origin through expatriate professional networks. This paper aims to explore the professional links that migrant health workers from sub-Saharan African countries living in five African and European destinations (Botswana, South Africa, Belgium, Austria, and the United Kingdom) have to their countries of origin.

**Design:**

Qualitative interviews were conducted with migrant doctors, nurses, and midwives from sub-Saharan Africa (*N*=66). A qualitative content analysis of the material was performed using the software ATLAS.ti.

**Results:**

Almost all migrant health workers have professional ties with their countries of origin supporting health, education, and social structures. They work with non-governmental organizations, universities, or hospitals and travel back and forth between their destination country and country of origin. For a few respondents, professional engagement or even maintaining private contacts in their country of origin is difficult due to the political situation at home.

**Conclusions:**

The results show that African migrant health workers are actively engaged in improving living conditions not only for their family members but also for the population in general in their countries of origin. Our respondents are mediators and active networkers in a globalized and transnationally connected world. The research suggests that the governments of these countries of origin could strategically use their migrant health workforce for improving education and population health in sub-Saharan Africa. Destination countries should be reminded of their need to comply with the WHO Global Code of Practice for the international recruitment of health professionals.

The global inequitable distribution of healthcare workers contributes to the malfunction of health services in many low income countries, while several high- and middle-income countries profit from the migration of skilled health workers. The loss of healthcare workers is therefore catastrophic to the already under-resourced health systems of many poor countries (1–6). The underlying determinants of migration are manifold and best explained by the concept of push and pull factors (7, 8). Migration of health workers is especially severe in sub-Saharan Africa (SSA). In many SSA countries the vacancy rate of doctors and nurses in the public health sector amounts to more than 50% (5, 9, 10). The brain drain has far-reaching consequences regarding equity in healthcare on a global scale (11, 12). So far, responses to migration have focused on restrictive work contracts or immigration policies; however those measures failed to stem the exodus and mostly targeted the pull factors (9, 13).

Whereas the restrictive migration policies for individual health workers in destination countries have been extensively and critically discussed, there is little research regarding the possibilities that countries of origin have for mitigating the negative consequences of brain drain (14). Kollar and Buyx (14) suggested a three-step strategy for handling the health-worker brain drain debate: first, destination countries have to direct more resources to countries of origin to compensate them for their human resource loss. Second, destination countries should scale up domestic training of an adequate health workforce; third, only when the first and second conditions have been solved can staff retention measures by countries of origin be discussed. The model is based on the assumption that the majority of health workers migrate because of bad working and living conditions in their countries of origin, an assumption that is supported by other research with migrant health workers (15–17). Admittedly pull factors such as economic wealth in the destination countries play an important role in international migration, and many countries train health workers (nurses and doctors) for the international market for generating remittances (e.g. Sudan, Cuba, Philippines) (9, 18). The result of such a policy can be that there are not enough health workers to serve the population in the country of origin and therefore interventions in staff retention should be urgently discussed.

Although emigration of highly skilled persons like health workers can be considered a clear human resource loss for the country of origin and a net gain for the destination country, countries of origin can also benefit from their populations in diaspora. Whereas repatriation of migrant persons is difficult for countries of origin, return of knowledge and skills seems more feasible (9). Migration is a dynamic process including a variety of networking activities by migrants in source and destination countries (9). Emigration does not necessarily mean that the person cuts all ties with the country of origin; on the contrary, migrants most often have ongoing contact with family, friends, and colleagues and actively engage in improving living conditions for people in their countries of origin (15, 19–22). Many countries have already recognized the strength that their diaspora can have. In Korea and Taiwan, for example, the government strategically supports diaspora organizations of highly skilled persons all over the world with significant benefit to the countries of origin (9). Migrants are known to support (extended) family members back home with remittances and to give other forms of financial backup to communities, such as investment in businesses or infrastructure (e.g. construction of houses or wells) (23–26). However, diaspora engagement can also go beyond financial support.

In general, migrant health workers belong to a mobile middle class with a high level of education, with more economic and social resources at their disposition than lower skilled migrants, who often have a difficult residence status in the destination country and are not as mobile (27, 28). This increased mobility can allow migrant persons to stay connected to their countries of origin. Therefore, migrant health workers’ activities in support of their countries of origin can be very diverse (29–32). Yet studies about positive effects (besides remittances) of international health worker migration for the countries of origin are scarce.

Against this background, this paper aims to explore the professional links that migrant health workers from SSA countries living in five African and European destinations (Botswana, South Africa, Belgium, the United Kingdom, and Austria) have to their countries of origin.

## Methods

### Study design

This study was conducted as part of a larger EU-FP7 funded research project on Human Resources for Primary Healthcare in Africa (HURAPRIM; www.huraprim.ugent.be) involving eight partner countries. Semi-structured interviews with 88 female and male migrant health workers were conducted in five of the eight project partner countries, namely Botswana, South Africa, the United Kingdom, Belgium, and Austria between July 2011 and April 2012. The other three partner countries (Mali, Uganda, and Sudan) did not participate in that study because they are foremost countries of origin and not destination countries. For the purpose of this paper only the interviews with doctors, midwives, and nurses (*N*=66; 25 nurses/midwives and 41 doctors) were analysed. The other 22 interviews were conducted with medical technicians, laboratory assistants, or other health professionals. For better comparability, we decided to exclude them from the analysis of this particular paper.

The interviews were conducted in English, Dutch, French, or German using a semi-structured interview guide. The detailed process of guideline development, recruitment process, and data collection have been described in depth elsewhere (15, 17, 33, 34). Therefore we only briefly mention the relevant points for this paper here.

The interviews took place in the United Kingdom (*n*=12), Belgium (*n*=14), Austria (*n*=10), Botswana (*n*=15), and South Africa (*n*=15). The three main research questions in the interview guide were as follows: 1) personal experiences and reasons for migration of health workers, 2) continued links with their countries of origin, and 3) future (migration) plans. This paper focuses on the question regarding the professional links to the countries of origin of the migrant health workers.

### Recruitment and data collection

The recruitment and data collection are extensively described in other studies (15, 17, 33, 34). For this study we therefore limit ourselves to presenting the most important steps of data collection and recruitment. The inclusion criteria for participants were as follows: 1) born in SSA (one interviewee was not born in SSA, but had been trained in South Africa); 2) received professional training in medicine or nursing in SSA. In all countries, locally adapted strategies for purposeful sampling were applied to find study participants. Letters for participation were sent to migrant organizations, nursing homes, local health service employers, hospitals and other health organizations, and unions. District family doctors were contacted for lists of names in South Africa. Calls were circulated in online forums. One of the more successful strategies in all countries was the snowball technique.

Researchers with extensive knowledge in qualitative methods conducted the interviews. They lasted between 30 and 90 min and took place at the respondent's preferred place, usually at work or at home. Interviews were recorded and transcribed verbatim by the interviewers or by other research team members.

### Data analysis

The 66 interviews analysed for this paper were imported into the software ATLAS.ti for qualitative content analysis. For the purpose of this study, one researcher (SW) deductively coded and analysed the interviews according to the research question formulated in the interview guideline, ‘Are you linked to your home country professionally?’ (35–37). After reading through the answers of the interviews to that particular question, four codes were defined that are also reflected in the subtitles of the results: 1) working with non-governmental organizations (NGOs) and in primary healthcare projects; 2) registration or practising at home; 3) scientific work in home country; 4) difficulties for diaspora engagement. These codes were further summarized and analysed together with another researcher with extensive knowledge in qualitative methods who was also familiar with the interview material (RK) (38). We present the results by including many original excerpts from the interviews; by doing so we aim to give our research partners a strong voice. After several drafts of the manuscript with the co-authors who conducted or were familiar with the content of the interviews, the results were finalised and are presented below.

### Ethical statement

The study was approved by the ethics committees of each partner university: Ghent University, Belgium (Ref.: 2011/552), University of Oxford, UK (MSD/IDREC/C1/2011/96), University of Botswana (PPME 13/18/1 VII (368), Medical University of Vienna, Austria (EK-Nr: 989/2011), and the University of the Witwatersrand, South Africa (M111122). The data produced during the project is strictly confidential, and interviewees are anonymous in all transcripts and analyses. We deleted the countries of origin of the respondents in the verbatim citations and named only the destination country in order to maintain anonymity. Prior to the interviews, all participants were informed about the HURAPRIM project and its objectives, as well as the purpose of the interviews. Written informed consent was obtained from all participants.

## Results

### Sample characteristics

The respondents (*N*=66) came from 18 different countries of origin (see Table 1) (15, 17, 33, 34). There were 25 nurses/midwives and 41 doctors. In total, we interviewed 35 female (13 doctors/22 nurses) and 31 male participants (28 doctors/3 nurses). Some countries of origin were overrepresented, for example, South Africa and the Democratic Republic of Congo (DRC). The majority of the respondents had lived in their destination country for more than 5 years, and most were between 30 and 50 years old.

**Table 1:** Respondents by country of origin

Country of origin	Nurses/midwives, *n*	Doctors, *n*
Angola	1	0
Cameroon	0	1
Congo Brazzaville	1	0
Democratic Republic of Congo	2	15
Gabon	0	1
Ghana	0	1
Guinea	0	2
Ivory Coast	1	1
Nigeria	2	4
Rwanda	1	2
Senegal	0	1
Somalia	0	1
South Africa	9	6
Sudan	0	1
Tanzania	0	1
Uganda	1	2
Zimbabwe	1	1
Zambia	7	1
Total	25	41

One important feature of the migrant health workers we interviewed was that most of them were very mobile and travelled back and forth between destination country and country of origin (or other countries). Some travelled every few months, when the country of origin was close by (e.g. from Zambia to Botswana) or twice a year when the country was far away (e.g. from a European country to Ivory Coast, DR Congo, or South Africa). However, for some respondents it was not possible to go back to their country of origin, as they left the country for political reasons and some were still not able to go back due to political conflicts. Almost all had professional ties with their countries of origin, supporting health, education, and social structures in their country of origin or in other SSA countries. Many respondents had founded or were working with NGOs in their country of origin. The projects were situated within public health or in the education sector. Several respondents still renewed their work registration as a nurse/midwife or doctor every year in their country of origin and some still practised in the health profession whenever they travelled back. In addition, some respondents engaged actively in university teaching in their countries of origin as visiting lecturers.

### Working with NGOs and in primary healthcare projects

Many of our respondents were engaged in working with NGOs from their countries of origin or in primary health or education projects. Some were collecting money or other resources for the organizations. One female nurse working in the United Kingdom, for example, founded an NGO that provides scholarships to orphans and was setting up a hospice in her country of origin. She is one example demonstrating that ties to the country of origin do not have to diminish after migration; on the contrary, during the interview the respondent explained various times what projects and vision she had for her country of origin and the various ways she is still connected.

Then in [country of origin], in my rural area I decided to set up this NGO, which is offering scholarships to the orphans. That NGO has been doing very well […] So because of that the community in my area decided to donate a land which is 350x100 acres […] Yes so there we're going to be having a hospice, we're going to be having an HIV and AIDS academy provided we're getting funds. (Female nurse, UK, Int 39)

A doctor living in Austria links doctors from the destination country to her country of origin. They work in rural areas, training health workers in family planning and primary healthcare, and try to build a clinic. The migrant doctor functions as the link between her home and destination country. A very active nurse living in Belgium has refugee projects in more than one African country; she founded an association that trains refugees in different professions.

We have projects there and here. For the time being we work in [an SSA country] because there are also refugees there. […] There is someone there to welcome them. Our association gives classes there, for them to be able to work. And in the hospital in [country of origin] I am still in contact with the doctor. (Female nurse, Belgium, Int 18)

In addition, during the interview she explained how the feeling of being a migrant herself woke the urge to help other migrants, as well. Many respondents felt they owed something to their country of origin because they trained there and their conscience often bothered them because they decided to leave. The connection to the country of origin and the urge to contribute is shown in the following quote from a doctor living in Belgium as well. He explains that he feels attached and obliged to give something back to his country of origin.

I work privately and with associations in [country of origin], for example we have two projects where we work with schools. […] We built a library that is free for everyone but especially for schoolchildren. […] For six years I got a scholarship from the government, and it is not only that; as a [citizen of the country of origin] I feel I have to do something for my country. (Male doctor, Belgium, Int 3)

Another doctor living in Austria is planning an NGO with a colleague who still lives in the country of origin. A nurse living in Belgium is working with a medical NGO and stresses her willingness to participate in operations in African countries. Ongoing connections are very important to all respondents.

The diaspora can help, for example I am planning an NGO with a colleague from Africa. For one year now always if we have the time we discuss it, how we can help. (Female doctor, Austria, Int 2)

A female doctor in the United Kingdom is working in the area of maternal health in her country of origin; she fulfils a role as a mediator between different organizations and her country. As she knows the structures in her country of origin well, she also knows which steps are necessary to be able to do NGO work:

One or two people in these organizations have a charity, they really put a lot of time into [it], so we [her work place] are trying to link it up to some very rural areas in [country of origin]. In fact my boss is in [country of origin] at the moment trying to see how [it could work]. […] It's maternal health. So it's promoting, improving motherhood facilities and training for health workers; I've linked up maternity worldwide with the Ministry of Health [in country of origin]. (Female doctor, UK, Int 38)

The following example of a male doctor living in the United Kingdom shows a different aspect of diaspora engagement: he is involved in various healthcare projects in his country of origin, always moving back and forth between his destination country and his country of origin. For him circular migration has become a lifestyle where the destination country is the home base and the country of origin is the place where he is able to start businesses and to develop healthcare structures.

At the moment I've got two start-up businesses in [country of origin] in healthcare that are running which I started during the MBA; I'm very happy to be in [country of origin], very happy to work, very happy to build things; but to be honest I'm only happy as long as I can get out of the country whenever I want and know that I could work in the UK or somewhere else and that my asset base will be safe and I would not want to raise a family in [country of origin]. (Male doctor, UK, Int 21)

### Registration or practising at home

Many respondents were still registered as nurses, doctors, or other health staff in their country of origin, paying for their licence and renewing it regularly. In that way they are contributing financially to the maintenance of health structures. A cited reason was that they did not want to lose track of how the work processes were developing at home, and they wanted to hold on to the opportunity of going back to work there. Two respondents were still practising back home; whenever they travelled to their country of origin, they would work a few days in their field.

So far the only link is the registration because when I renew my licence here every year I have to go back home and renew it and pay for re-registration every year. (Female nurse, Botswana, Int 28)

As I said I did a lot of surgery and I have a few very good friends that I often have contact with, and whenever I go back to [country of origin] I still am registered with the Health Professions Council in [country of origin] so I still have [my] registration there. So I often go and assist in theatre still, whenever I go back to [country of origin] I always spend a day or two in hospital. (Male doctor, UK, Int 21)

The efforts to stay connected to the country of origin were diverse and the reasons were professional as well as private. Many respondents have family members back home and do not want to lose the link. In addition, the feeling of being more useful in their profession as health worker in their country of origin than in their destination country plays a role.

When I start getting closer to finishing my PhD I'll start making, try looking at those links and going and seeing people and looking for a position to specialise […] because [country of origin] is home, I've got family there. Because I feel that I can contribute there more than I can here, I feel that I've learnt skills here that I can take back. (Male doctor, UK, Int 24)

### Scientific work in country of origin

In some cases our respondents were teaching at universities in their countries of origin or planning to do so. Others had joint research projects with partners in their countries of origin and were interested in keeping those contacts. A female nurse went to South Africa to complete a higher nursing degree, to be able to teach and give lectures in her country of origin, because there was no possibility of higher nurse education there. She wanted to gain skills to feed back into her country of origin. There was another example from a nurse living in the United Kingdom. She wanted to do her research in her country of origin, trying to use the skills she acquired in her studies to answer the question of how it would be possible to increase the number of students in nursing in her country of origin. Those two are good examples of brain circulation and the range of possibilities that migration can be used for to improve global health.

My plan is to do it [research] in [country of origin] and within the nursing institutions of [country of origin]. […] Yes, around issues of education because I'm looking at the clinical skills lab because like currently the nursing colleges in [country of origin] they have been requested to increase the number of students. (Female nurse, UK, Int 33)

For more senior doctors or nurses, the links with the country of origin were kept through ongoing research projects. One male doctor, for example, still had obligations concerning the writing of papers with colleagues or new research proposals. This doctor brought his former network to the destination country Botswana and it could easily be used as an opportunity to strengthen bilateral collaboration. The migrant doctor could be viewed as the mediator in that process.

I still have continued links with [my] country of origin because there are projects that I used to do in [country of origin]. I'm still collaborating with these people […] and there are some research papers that we are writing because when I came to [the destination country] I still had to complete some research projects in the country of origin […] and I contacted my friends because I have written a proposal where I want to inform my country of origin. (Male doctor, Botswana, Int 27)

For another male doctor living in Austria, collaboration with a university in his country of origin is already becoming a reality. He travels back and forth between destination and country of origin and teaches there.

I already made contacts, I will probably be teaching at the university there the coming semester. Not only practical trainees – courses, seminars, workshops, twice a year for free, that I travel there for two weeks to give a seminar, for example. There is also another technological school […] where I will be teaching. (Male doctor, Austria, Int 3)

### Difficulties for diaspora engagement

For some of the respondents, professional engagement or even maintaining private contacts in their country of origin was difficult or not possible at all due to political reasons in the country of origin.

It is my country; I am strongly attached to it. But the problem is I cannot even put a foot there. It's sad. Someday I want to help the people over there, but now I can't. (Female nurse, Belgium, Int 14)

Today, the situation is still not like that that I can go back easily. There is political persecution. Now it is a little bit better but back in the days they even bugged telephone calls, opened letters. But even today when I talk I am not allowed to speak my mind fully. […] I can travel there and maybe nothing happens, but maybe they will accuse me of something that has nothing to do with the reality. (Female nurse, Austria, Int 6)

Two doctors living in South Africa also said that they were not allowed to travel to their countries of origin due to their precarious residence and work permit status.

No, because I am a refugee [I cannot travel home]. We applied for permanent residence, because we stay here more than five years. So we applied for permanent residence, we are waiting for them to reply. (Female doctor South Africa, Int 9)

I wanted to travel last year but I couldn't. The reason being that I applied for a new work permit and then there was this long period of waiting and I could not get my work permit. It didn't make sense for me to travel outside South Africa without a permit to return. If I go and then I come back, I will be deported from the airport and then it's going to be a long process you know with lawyers. (Male doctor, South Africa, Int 8)

However even when the respondents did not directly travel to their countries of origin, they tried to support people in their country of origin. For example, one female doctor travelled home for the last time in 2006 but when asked whether she (and her husband) supported people back home, she agreed and explained how she supported members of the family financially for study purposes:

I went there [in] 2006 and since [then] I have not been there. […] We do support people, even on my husband's side. Especially for people who want to study. When my mother was still there, because she was a widow, so I was like trying to send her some money. (Female doctor, South Africa, Int 7)

## Discussion

Our results show that African migrant health workers are actively engaged in improving living conditions not only for their family members back home but also for the general populations of their countries of origin or other African countries.

Most studies about migrant health workers underline the contribution they make to the destination country in terms of workforce building (15, 33, 34) or to the country of origin in terms of remittances (25, 26, 39–43). Here, we show that migrant health workers significantly contribute to the sending countries beyond the obvious link of remittances. Consequently, migrant health workers should be used much more as assets for the countries of origin. Our respondents are mediators and active networkers in a globalized and transnationally connected world. Whereas governments of destination countries fail to compensate the countries of origin for the loss of their highly needed workforce as suggested by Kollar and Buyx (14), migrants often step in and actively take the role of trying to compensate for the loss. Even though compensation of source countries is suggested in the WHO *Global Code of Practice on the International Recruitment of Health Personnel*, this issue is very complex. Kollar and Buyx (14) suggest it as one step toward resolving the global human resource crisis in healthcare. It is however not clear what such compensation would look like in reality. It is very complicated to calculate the loss of health workers for a society. Dovlo (44) suggests calculating ‘the costs of training and the loss of contribution to the GDP and taxes, the costs of illness/morbidity caused or aggravated by staff shortages, and costs arising from substituting less qualified staff or importing expatriates to fill the vacant posts’ (page 5). However, that is a large challenge because data on health worker migration is scarce in many sub-Saharan African countries, as well as in destination countries (4, 5). Moreover, many migrant health workers practise in the private health sector in the destination countries and legally binding compensation schemes from private healthcare seems even more complicated than in publicly funded healthcare (45). The only global political strategy up to the present seems to be the restriction of the migration of individual health workers from countries with shortages (46). In their *Framework Convention on Global Health* Gostin et al. (47) propose installing a more binding instrument than the WHO voluntary code [see also Mackey and Liang (48)]. The authors advocate for a compulsory compensation (financially and in form of workforce sharing programmes) from destination countries towards countries of origin, but in the framework of an international alliance.

Most of our respondents were working with or had founded NGOs focusing on health or primary care projects in their countries of origin. They felt indebted to their countries of origin and felt obliged to help, as they were once granted scholarships or training opportunities. All interview partners wanted to improve life in their countries of origin. Unfortunately, it was not possible for all respondents to go back, as some had left for political reasons. However, even those respondents supported NGOs financially and materially or worked in organizations in other African countries.

If the individual efforts of migrant health workers were better organized and supported by governments, the contributions of the diaspora populations could have a positive impact on the situation of human resources in many SSA countries (9). This possibility is particularly important since repatriation of the migrants is not realistic, and it is hard to compete with the economic conditions of most destination high- or middle-income countries. Many migrants also do not want to return (permanently) for private reasons such as raising children in the destination country, for example, factors known as ‘stick’ and ‘stay’ factors (7). One option for migrant health workers who cannot or do not want to travel back and forth would be to get engaged in online knowledge and skills transfer, which is known as ‘digital knowledge networks’ (49).

A UNESCO report outlines how countries of origin could benefit from their expatriate healthcare workers and suggests steps for the source as well as for the destination countries to engage with diaspora communities. The report recommends that countries of origin must make efforts to communicate with their diaspora and to create a friendly attitude toward their expatriates. In addition, it outlines the responsibility of destination countries to cooperate with and support their immigrant populations so that they can benefit their countries of origin (9).

Most of our respondents belonged to the middle class and many of them had assets such as good training and contacts with health workers in other countries; they spoke several languages and were familiar with other cultural backgrounds. Hence they had large amounts of social and cultural capital to offer (50). These resources should be supported and made available for improving global health in a sustainable way. Ankomah et al. (32), for instance, call for active engagement of the African diaspora in development plans and programmes. In Ghana, such a strategy has been fairly successful and Ghanaian migrants in the diaspora are involved in a variety of projects boosting the Ghanaian economy (51–53). Our findings support other recent work about migration that show that migration has become a middle-class phenomenon (28).

Destination countries can do more. The European Union adopted the ‘Global Approach to Migration’ that has as its second objective strengthening cooperation and exchanging best-practice models with SSA countries. However, it appears that the policy has mainly focused on reinforcing border controls by scaling up maritime operations (FRONTEX) and by making immigration for all kinds of non-EU migrants very complicated (13). The EU should work more toward fulfilling the goal of strengthening cooperation. The countries of origin could work on bilateral agreements and ask the destination countries to engage in exchange programmes for building capacity at universities or in the health system. There are also many suggestions in the WHO *Global Code of Practice on the International Recruitment of Health Personnel* (54). It tackles global health concerns, the cooperation of governments, as well as fairness and transparency in global health matters. The code is voluntary and it tries to establish a global architecture, including ethical norms and legal and institutional arrangements, to guide national action and multilateral cooperation to support sustainable health systems, protect the human rights of migrant health workers, and support health systems in low- and middle-income countries. Since its publication in 2010 there have been few studies on the impacts of the WHO Code of Practice. Edge and Hoffman (55) evaluated the impact of the code in Australia, Canada, the United Kingdom, and the United States. The majority of the stakeholders believed that the code did not have any significant impact on policies, practices, and regulations in their countries. Siyam et al. (56) found that it was difficult to engage multiple stakeholders – at the national and subnational levels and in the public and private sectors. Hence, more needs to be done on the level of awareness regarding the WHO Code of Practice. For example, migrant health worker organisations or diaspora organisations could contribute to enhanced awareness in destination countries of the need to comply with the Code of Ethics to compensate countries of origin and strengthen cooperation with them. The civil society–led advocacy initiative Health Workers for All and All for Health Workers^1^ in Belgium are intensively working on the implementation of the WHO Global Code of Practice in the European region and are an important stakeholder in that matter, as well.

The South African Department of Health released a new policy in 2006 regarding the employment of foreign health workers (57). The policy states that, in order to prevent negative consequences from brain drain, cooperation and regional partnerships should be strengthened. That could be an opportunity for countries of origin to engage in negotiations. The position of South Africa in the debate on human resources for health deserves more attention. South Africa is both an important source country for the United Kingdom and Australia and at the same time one of the most prominent destination countries for skilled health workers from other SSA countries. In 2010, the Health Professions Council of South Africa agreed on anti-brain-drain measures and stated that recruitment of health professionals from countries with shortages should not be encouraged. On the other hand there has been a memorandum of understanding in place between the United Kingdom and South Africa since 2010 restricting recruitment of health professionals from South Africa in the United Kingdom (58).

The use of a diaspora workforce is only one small step in scaling up efforts to improve the health of populations on a global scale and especially for countries in the southern hemisphere. Other quantitative and qualitative measures are needed and have already been discussed broadly (12, 47, 59–62).

The forms of support the migrant health workers attempt to give to their countries of origin need to be discussed critically, too. The fact that migrant health workers work or support NGOs in their countries of origin that are part of the private healthcare sector and often have vertical disease-centred approaches entails risks regarding equity in health (i.e. inequity by disease) (63). Furthermore, the fact that some respondents regularly renewed their work licence in their country of origin may have led to a misinterpretation of numbers of human resource in health in the countries of origin. These workers appear in the statistics but are not physically present. It is therefore even more important that the governments of the countries of origin actively cooperate with their health workforce in the diaspora in order to integrate their efforts into the national healthcare system.

Many countries of origin engage in the support of their expatriate communities already. The government of Nigeria, for example, significantly supports the organization Nigerians in the Diaspora^2^ and Egypt has several agreements with other countries that allow Egyptian doctors to take sabbaticals to go to Egypt for up to 3 years to work there. There is also a trend toward diaspora health workers forming professional expatriate associations in order to support their countries of origin in a more organized way. The Armenian expatriate community in the United States sponsors several educational initiatives in the Republic of Armenia. Such examples, especially those involving government leadership, could set an example for both source and destination countries to facilitate a repatriation of knowledge and highly needed healthcare skills (9).

### Strengths and limitations

The diversity of our sample is a strength, as it shows that almost all migrants engage in professional activities in their countries of origin or at least wish to do so regardless of their country of origin or destination. For a qualitative study the sample size of *n*=66 is very high, resulting in a lot of primary data. In addition, the study responds to a research need in healthcare: the need to involve target groups in research. Recruiting respondents for in-depth face-to-face interviews proved challenging, especially in countries where there are not many migrant health workers from SSA countries. Therefore the teams decided to employ a purposive sampling strategy, which involves the risk of reporting bias. The NGOs or projects that are presented in the narratives from the migrant health workers were not further specified and details such as the amount of resources directed to the diaspora engagement or the duration of work involvement in the countries of origin are not always clear. Due to the variety of destination and countries of origin of the respondents it is difficult to generalize findings or to show patterns of diaspora engagement for one specific destination or country of origin.

## Footnotes

1
www.healthworkers4all.eu/home/

2
www.nidoamerica.org/ (one example; there are more in other countries, such as in Europe)
